# Process evaluation of a breastfeeding support intervention to promote exclusive breastfeeding and reduce social inequity: a mixed-methods study in a cluster-randomised trial

**DOI:** 10.1186/s12939-024-02295-0

**Published:** 2024-10-08

**Authors:** Henriette Knold Rossau, Anne Kristine Gadeberg, Katrine Strandberg-Larsen, Ingrid Maria Susanne Nilsson, Sarah Fredsted Villadsen

**Affiliations:** 1 Section of Social Medicine, Department of Public Health, University of Copenhagen, Øster Farimagsgade 5, 1353 Copenhagen, Denmark; 2 Section of Epidemiology, Department of Public Health, University of Copenhagen, Øster Farimagsgade 5, 1353 Copenhagen, Denmark; 3 The Danish Committee for Health Education, Classensgade 71, 5, 2100 Copenhagen, Denmark

**Keywords:** Complex interventions, Process evaluation, Cluster-randomised trial, Implementation, Breastfeeding, Health visiting, Infant health, Socioeconomic factors, Delivery of health care, Public health

## Abstract

**Background:**

Breastfeeding is a powerful public health intervention that produces long-term health benefits. However, in high-income countries such as Denmark, breastfeeding rates are suboptimal and unequally distributed across socio-economic positions. The ‘Breastfeeding – a good start together’ intervention, to promote longer duration of exclusive breastfeeding and reduce social inequity, was implemented in a cluster-randomised trial during 2022–2023 across 21 municipalities in two Danish regions. A process evaluation was conducted to assess the implementation, mechanisms of impact, and possible contextual factors affecting the intervention.

**Methods:**

The study was guided by the Medical Research Council’s guidance for conducting process evaluations and employed a mixed-methods approach in a convergence design. Quantitative data: contextual mapping survey (*n* = 20), health visitor survey (*n* = 284), health visitor records from 20 clusters and intervention website statistics. Qualitative data: dialogue meetings (*n* = 7), focus groups (*n* = 3) and interviews (*n* = 8).

**Results:**

Overall, the intervention was delivered as planned to intended recipients, with few exceptions. Health visitors responded positively to the intervention, noting that it fitted well within their usual practice and enhanced families’ chances of breastfeeding. Mothers expressed having received the intervention with few exceptions, and reacted positively to the intervention. Although health visitors were concerned about the potential stigmatisation of mothers receiving the intensified intervention, none of the interviewed mothers felt stigmatised. Contextual factors impacting the intervention implementation and mechanisms included staff and management turnover, project infrastructure and mothers’ context, such as resources, social networks and previous experiences. The overall fidelity of the intervention delivery was high.

**Conclusions:**

Health visitors and families responded well to the intervention. Interventions aimed at enabling health care providers to deliver simplified and structured breastfeeding support, in alignment with support provided in other sectors of the health care system, may increase breastfeeding rates and reduce social inequity in breastfeeding, even in international contexts.

**Trial registration:**

Clinical Trials: NCT05311631. First posted April 5, 2022.

**Supplementary Information:**

The online version contains supplementary material available at 10.1186/s12939-024-02295-0.

## Introduction

On a global level, breastfeeding is a powerful public health intervention that produces long-term health benefits and is a convenient, cost-effective and optimally nutritional food [1, 2]. In high-income countries, breastfeeding rates are suboptimal [3] and distributed inequitably across socio-economic positions in favour of more advantaged groups [4]. The same applies in the Danish context [5]. Mothers ask for accessible and individual support from health professionals [6] which has been underscored as crucial for duration and exclusivity of breastfeeding [7]. On this basis, a breastfeeding support intervention: ‘Breastfeeding – a good start together’, was rolled out during 2022–2023 in a sample of 21 Danish municipalities (clusters) [8] – henceforth referred to as ‘the Breastfeeding Trial’.

In the Breastfeeding Trial, health visitors in the intervention arm (*n* = 11 clusters) received education to provide individualised breastfeeding support to new families, based on current evidence and theories such as self-efficacy, tailoring, and attributional retraining [9], and aligned with the breastfeeding support implemented at hospital level [10]. The aim of the intervention was to strengthen breastfeeding support and increase the proportion of women accomplishing their breastfeeding goals. An additional hypothesis proposed that delivering a higher dose of the intervention through proactive telephone calls (termed: ‘intensified intervention’) to families with young mothers and/or low educational attainment could help reduce social inequity in breastfeeding. In a pre- and post-test study it was documented that the education programme enhanced health visitors' knowledge, action competence, and self-efficacy related to breastfeeding support [9]. The present study is a mixed-methods, systematic process evaluation of the Breastfeeding Trial prior to the trial effectiveness evaluation.

**Fig. 1:**
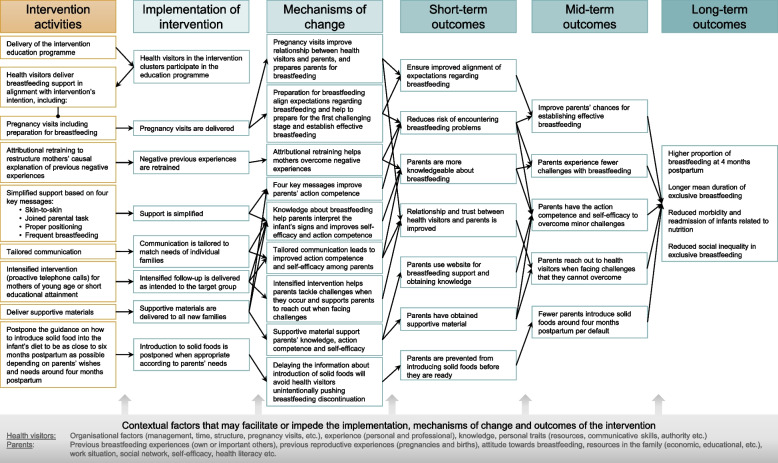
Programme theory depicting how the intervention activities are hypothesised to produce outcomes via implementation, mechanism of change and short- and mid-term outcomes

Complex interventions like the Breastfeeding Trial are likely to reflect many causal assumptions. Identifying and stating these assumptions, or ‘programme theories’, is vital if process evaluation is to focus on the key uncertainties that needs addressing, and hence advance the understanding of the implementation and functioning of the intervention [11]. The goal of a process evaluation is to illuminate the pathways linking an intervention to the outcomes produced in the end [12]. It may help reveal unanticipated consequences [13] and shed light on the intervention’s effectiveness and potential for scalability [12]. The causal assumptions behind the intervention are presented in the programme theory of the Breastfeeding Trial in Fig. 1.

To guide the process evaluation of the Breastfeeding Trial, we drew on the Medical Research Council (MRC) guidance from 2015 [12]. From this framework, we analysed the following three elements: 1) ‘Implementation’ (implementation process and delivery (dose, adaptations and reach)), 2) ‘Mechanisms of impact’ (participant responses to and interactions with the intervention, and unexpected pathways and consequences), and 3) ‘Context’ (factors that affect implementation, mechanisms and outcomes) [12]. Overall fidelity of the delivered intervention was assessed by integrating findings of the above three elements. By exploring the intricacies of the implementation of the Breastfeeding Trial, this study will contribute to examine the interplay between intervention components, implementation strategies and intended outcomes, to facilitate a deeper understanding of how and why the intervention produces effects, drawing into account contextual facilitators for and barriers to delivering the intervention. The findings from this study will inform future, planned evaluations of the trial [8], and essentially assist policymakers and practitioners in deciding on potential scale-ups of the intervention; however, the findings may also add valuable insights to other breastfeeding interventions or community-based interventions.

### Aim and objectives

We aimed to: 1) assess the implementation of the intervention by examining processes, dose, reach and adaptations; 2) explore the intervention’s mechanisms of impact, including responses and interactions and unexpected consequences; 3) identify contextual factors acting as barriers to or facilitators for the intervention; and 4) evaluate fidelity to the intended intervention. We addressed this in specific research questions, informed by the programme theory (Fig. 1). The research questions were as follows:

- *Implementation*
  - Was the intervention implemented and delivered as planned?
    - To whom?
    - What elements?
    - What adaptations were made?

- *Mechanisms of impact*
  - What were the reactions to and interactions with the intervention?
    - Among health visitors
    - Among families
    - Were there any unintended effects related to the intervention?

- *Context*
  - What contextual factors were important for the intervention to be implemented and delivered as planned?
    - What impeded or facilitated the delivery of the intervention and the intervention

- *Fidelity*
  - In an overall assessment, how did the interplay between the intended intervention, its implementation, the mechanisms of impact and the context affect the fidelity of the flexible, individually tailored intervention?

## The framework of the intervention

### Setting

In the present trial, the cluster units are municipalities with the control arm consisting of 10 clusters and the intervention arm consisting of 11 clusters. Municipalities are local areas of government with an array of responsibilities, including primary health care and prevention for children, under which the health visiting programme falls [14]. In Denmark, all mothers and their newborn infants are discharged from hospital after birth to a follow-up programme delivered by health visitors employed in health visiting programmes of the municipalities. The health visiting programme mainly consists of home-visits, is a tax-based offer and is largely accepted by more than 97% of new families [15]. Health visitors are registered nurses with an additional 18-month post-graduate education in promoting healthy families [16], including provisioning of breastfeeding support. In this setting, the present intervention was implemented. The average number of births per year in 2022 and 2023 when the intervention was tested sums to a total of 5,225 in the 11 intervention municipalities [17].

### The intended intervention and implementation

The intervention focussed on a strengthened breastfeeding support and included an 18 h skills education programme for health visitors covering breastfeeding physiology, breastfeeding support and tailoring of communication, among other things. Delivering a pregnancy visit to ensure parents’ breastfeeding preparation was part of the intervention. To support the intervention, materials were developed, including a dialogue sheet, a postcard, a pamphlet, and a website with guiding videos, podcasts and quizzes. Furthermore, the usual care four-month visit with guidance on how to introduce solid foods in the infant’s diet was to be postponed where possible to prolong exclusive breastfeeding. Thus, the ordinary four-month visit was replaced by a telephone call in which the individual parents’ needs regarding the introduction of solids were assessed. Health visitors in the intervention clusters were instructed to hand out the supportive materials to new families and provide simple, evidence-based breastfeeding support (Fig. 1). The intervention has been described in detail elsewhere[8], as has the education programme [9]. The four key messages in the intervention: 1) breastfeeding as a joint parenting task, 2) skin-to-skin contact, 3) proper breastfeeding positioning and 4) frequent breastfeeding, are evidence-based and have previously produced results on breastfeeding rates in the ‘Less is More’ trial carried out in Danish maternity wards [18, 19]. It was subsequently politically decided to implement ‘Less is More’ throughout Danish maternity wards [10]. The proactive telephone calls for mothers who are young or have low educational attainment, were inspired by an intervention study that found frequent telephone calls for mothers with a pre-pregnancy BMI ≥ 30, whom often have difficulties breastfeeding, to be effective in prolonging breastfeeding [20].

Invitation to participate in the Breastfeeding Trial was addressed to managers of the health visiting programmes in the municipalities in the North Denmark Region and Region of Southern Denmark. In municipalities accepting participation, one or two local project representatives (depending upon numbers of newborns in the municipality) were appointed, with financial compensation from the project funds, to act as day-to-day project coordinators. Monthly dialogue meetings were held between intervention developers and local representatives from intervention clusters, during which issues regarding implementation and the intervention could be discussed. The dialogue meetings underpinned the implementation and were continually held from April 2022 until September 2023. Reflections from the dialogue meetings gave rise to a shorter, online brush-up course being delivered in May 2023, to introduce to the intervention health visitors who were employed in the intervention clusters *after* the education programmes had been delivered in March 2022.

## Materials and methods

A mixed-methods design [21] was planned to comprehensively answer our research questions, and the MRC guidance for conducting process evaluations directed our assessment of the implementation [12].

The CONSORT statement: extension to cluster randomised trials [22] and the Standards for Reporting Qualitative Research (SRQR) [23] guided the study reporting.

### Process evaluation terms and definitions

#### Implementation

According to Moore et al. [13], implementation can be measured by implementation process and what is delivered (dose, adaptations and reach). The implementation process refers to *“… the structures, resources and mechanisms through which delivery is achieved*” [13](p. 8), and delivery refers to among others, dose (the quantity of the delivered intervention), reach (whether the intended audience has encountered the intervention) and adaptation (the changes made to the intervention to improve contextual fit) [13].

#### Mechanisms of impact

Mechanisms of impact refers to “*the intermediate mechanisms through which intervention activities produce intended (or unintended) effects*” [13] (p. 8). These include participant responses and interactions with the intervention, and unintended pathways and consequences [13].

#### Context

Context refers to factors that are “*… external to the intervention which may influence its implementation, or whether its mechanisms of impact act as intended*” [13] (p. 8). Contextual factors may influence the delivery and functioning of the intervention, hence either impede or strengthen the implementation, delivery and mechanisms of the intervention and thereby its effects [12].

#### Fidelity

According to MRC guidance, assessing fidelity (whether the intervention was delivered as intended) is part of evaluating implementation [12]. However, because the intervention is flexible and tailored to individual needs, measuring how it plays out is complex. Fidelity will vary between individuals. In this study, we will summarise the results and assess fidelity by examining the interplay between the intended intervention, its implementation, mechanisms, and context.

### Data sources

The data sources are described in detail below, divided into whether collection was completed throughout *all project clusters* or in *intervention clusters only*. A full list of data sources and which research questions they inform is provided in Additional File 1.

#### Data collected in all clusters

##### Organisational survey

In early 2023, an organisational survey was distributed electronically to managers of the health visiting programmes in all municipalities. Because one intervention cluster dropped out immediately after the education programme, this cluster was omitted from the survey. The survey focused on the local and organisational context, and included the following themes: 1) management and size of the health visiting programme, 2) organisation of visits in the health visiting programme, prespecified according to the recommendations by the Danish Health Authority, 3) estimation of the proportion in need of extra visits, and 4) organisation of staff, team meetings and conditions possibly impacting staff resources, and the managers were asked to reply based on their current programme. A full overview of the questions included in the questionnaire can be found in Additional File 2.

##### Health visitor survey

Electronic questionnaires were distributed to all health visitors employed in the participating municipalities: 1) at baseline: prior to education in December 2021, and 2) at follow-up: in October 2022 six months after education. Themes in the survey questionnaires were: 1) background and education, 2) breastfeeding support, attitudes, and practices in relation to breastfeeding support and self-reported relationships with families. Additionally, health visitors in the intervention arm were asked about their experience with the intervention, the education programme and the intervention material (for example, experiences with and attitudes towards) at follow-up. Full overview of the questions included in the surveys, timepoints for distribution and recipients can be found in Additional File 3.

##### Health visitor records

Average visits and telephone calls per mother-infant dyad per month were collected from the health visitor records in each municipality.

#### Additional data from clusters in the intervention arm only

##### Website logins

Use of the website was monitored by simple analytics providing number of logins with few options available for data extraction and no option for filtering data on municipality. Data accessible were most used unit of access (smart telephone vs. computer), average time spent during visits and most popular webpages on the website. Data from the intervention website were extracted of a one-year period from 1 June 2022 to 1 June 2023.

##### Focus groups with health visitors

Three semi-structured, online focus groups were conducted. Two health visitors from each of the municipalities in the intervention arm, not already appointed as local representative, were invited. HKR moderated the focus groups, assisted by AKG as an observant.

##### Interviews with parents – predominately mothers

Eight semi-structured interviews with mothers were conducted, one including both parents, and sampled from two of the intervention sites. Interviews were conducted either face to face in parents’ home (*n* = 5) or over the telephone (*n* = 3), depending on the interviewees’ preferences. The individual interviews were conducted by HKR.

##### Dialogue meetings

Minutes and observations from the first seven of a total of 13 dialogue meetings informed the present process evaluation, during which a representative from the evaluation team participated in an observer role.

### Analytical approach

#### Quantitative data

Quantitative data were analysed descriptively and presented graphically. For comparisons of means, frequencies and medians across trial arms, Welch’s *t*-test, Chi-square, Fisher’s Exact test or one-way ANOVA on ranks test were used. Analyses were done using SAS statistical software [24].

#### Qualitative data and analytical approach

Qualitative data were analysed using Systematic Text Condensation (STC) focusing on participants’ expressed experiences [25]. HKR, AKG and SFV discussed the transcripts and the initial coding. Coding was carried out using NVivo 14 software [26].

#### Integration of findings

Initial thorough analyses were conducted for each data source individually. Subsequently, using a mixed-methods convergent design, integration of findings data sources was completed to compare the convergence, divergence or complementarity of the findings, thereby gain an in-depth and comprehensive answer to the research questions [21]. The aim was to provide a general impression of the fidelity of the intervention in the context in which it was implemented. To improve readability, the results are structured into the three categories of the MRC’s guidance: implementation, mechanisms and context. However, given the overlap between these categories the separation of themes into categories remains somewhat fluid to avoid repetition and fragmentation.

## Results

### Response rates and participants

All managers of the project municipalities (*n* = 20) responded to the organisational survey. Data are provided in Table 1.

**Table 1:** Organisational contextual factors reported cross-sectionally in March 2023, distributed on trial arms

	Control clusters (*n* = 10)	Intervention clusters (*n* = 10)	
	Mean (range)	Mean (range)	*p*-value*
Number of newborns in 2021	627 (195–2390)	570 (348–1229)	0.724
Number of newborns in 2022	582 (138–2200)	518 (310–1150)	0.579
Shift in management since project start in 2021, n (%)	3 (30)	5 (50)	0.240
Number of health visitors employed, median (IQR)	15 (12–17)	13 (10–18)	0.868
Proportion of staff turnover during 2022, %	12 (0–28.6)	18 (0–50)	0.355
Number of IBCLCs employed, median (IQR)	2 (1–2)	2 (1–3)	0.653
Proportion reporting appointed specific health visitors handling the care of special groups, for instance young mothers, n (%)	7 (70)	4 (40)	0.150
Frequency of team meetings, n (%)			0.053
Weekly	3 (30)	4 (40)	
Biweekly	4 (40)	1 (10)	
Monthly	3 (30)	5 (50)	
Proportion of municipalities offering pregnancy visits, n (%)			
For primiparous families	8 (80)	9 (90)	0.395
For multiparous families	6 (60)	8 (80)	0.244
Time allocated to pregnancy visits, minutes	78 (60–115)	80 (60–120)	0.977
Time allocated to first visit after birth, minutes	53 (30–90)	62 (30–90)	0.533
Estimated proportion of families in need of extra needs-based visits, %	36 (20–63)	29 (0–50)	0.512
Estimated proportion of families declining the health visiting programme in 2022, %	1 (0–5)	1 (0–2)	0.873

*Abbreviations: IBCLC* International Board Certified Lactation Consultant, *IQR* Interquartile Range

^*^P-values are calculated using Wilcoxon rank sum test for categorical data and skewed continuous data, Fisher’s exact test for frequencies and *t*-test for normally distributed continuous data

**Fig. 2:**
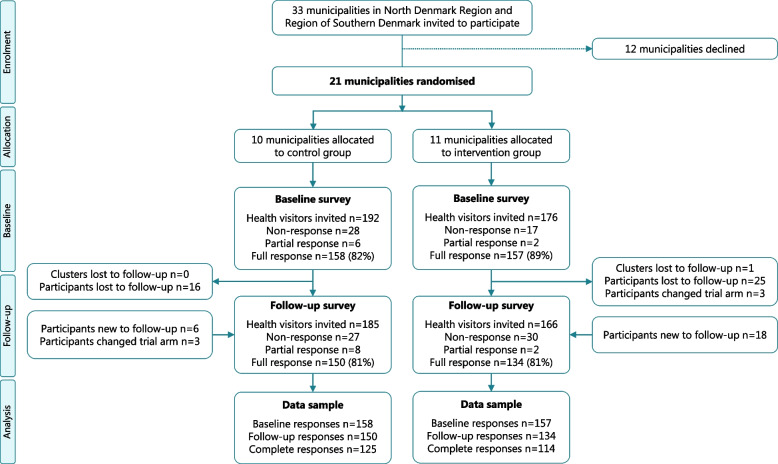
Flow diagram of heath visitor survey based on CONSORT

The health visitor survey was distributed to 368 health visitors at baseline, and 351 health visitors at follow-up. Figure 2 presents a detailed flow diagram of the inclusion and attrition of participants in the survey waves. The response rates were high in both trial arms (> 80%). Three health visitors changed trial-arm. Analyses of health visitor survey data are mainly based on follow-up responses (control arm *n* = 150, intervention arm *n* = 134), except for Fig. 6b in which self-reported assessments of breastfeeding support are based on complete responses in the intervention arm (*n* = 114).

Data from health visitor records were obtained from all municipalities (*n* = 20).

**Fig. 3:**
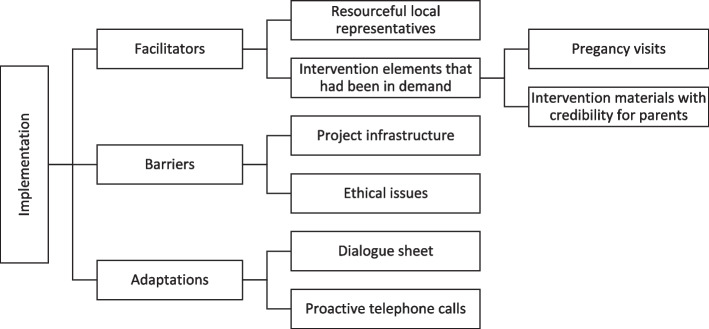
Coding tree – dialogue meetings with local representatives

In dialogue meetings, all local representatives participated if possible (*n* = 13). In the seven meetings analysed, 12–13 local representatives participated in each. A coding tree of the analysis can be found in Fig. 3.

**Fig. 4:**
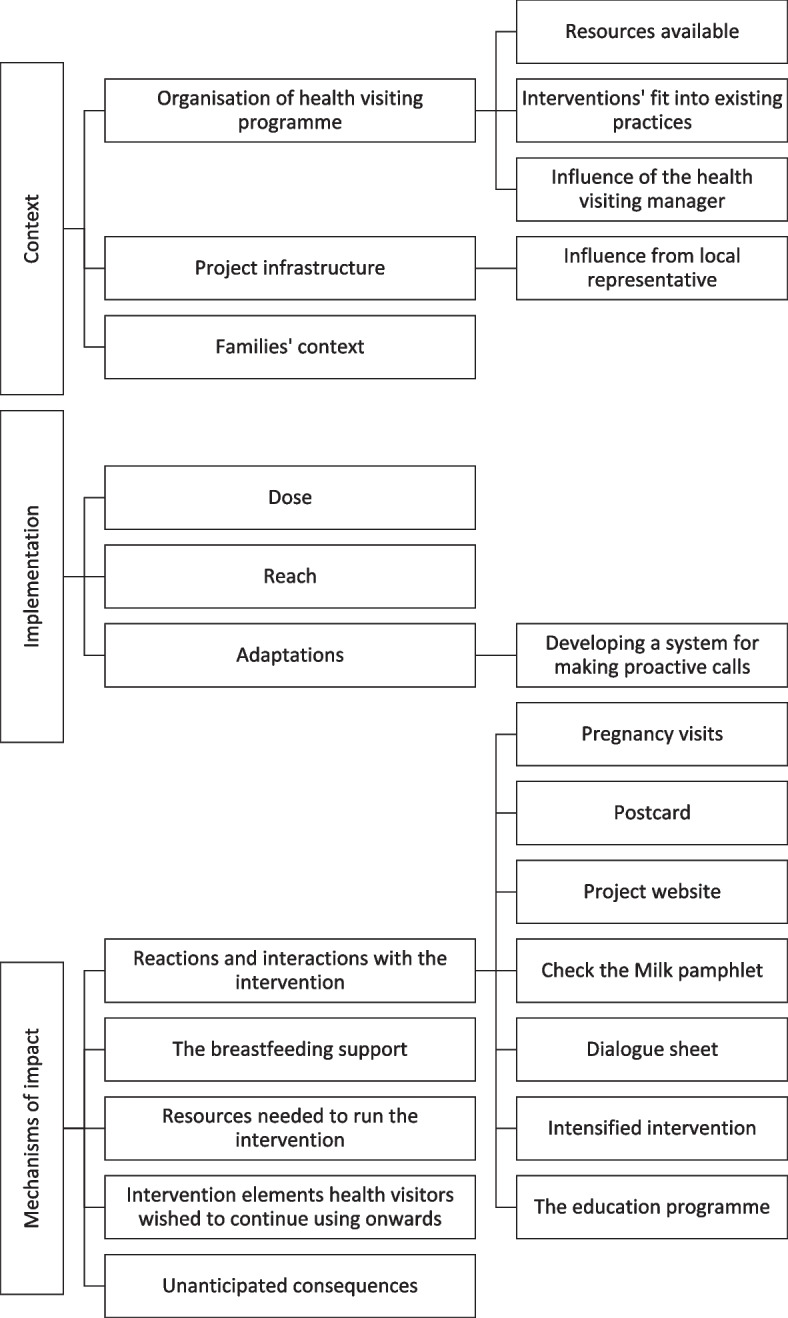
Coding tree – focus groups with health visitors

Sixteen health visitors participated in the three focus groups, with four to seven participants in each. Four out of 10 municipalities participated with just one participant. Participants had between six months and 31 years of experience as a health visitor, two held International Board Certified Lactation Consultant (IBCLC) qualifications, and one had not participated in the education programme (data not shown). A coding tree of the analysis of focus groups can be found in Fig. 4.

**Fig. 5:**
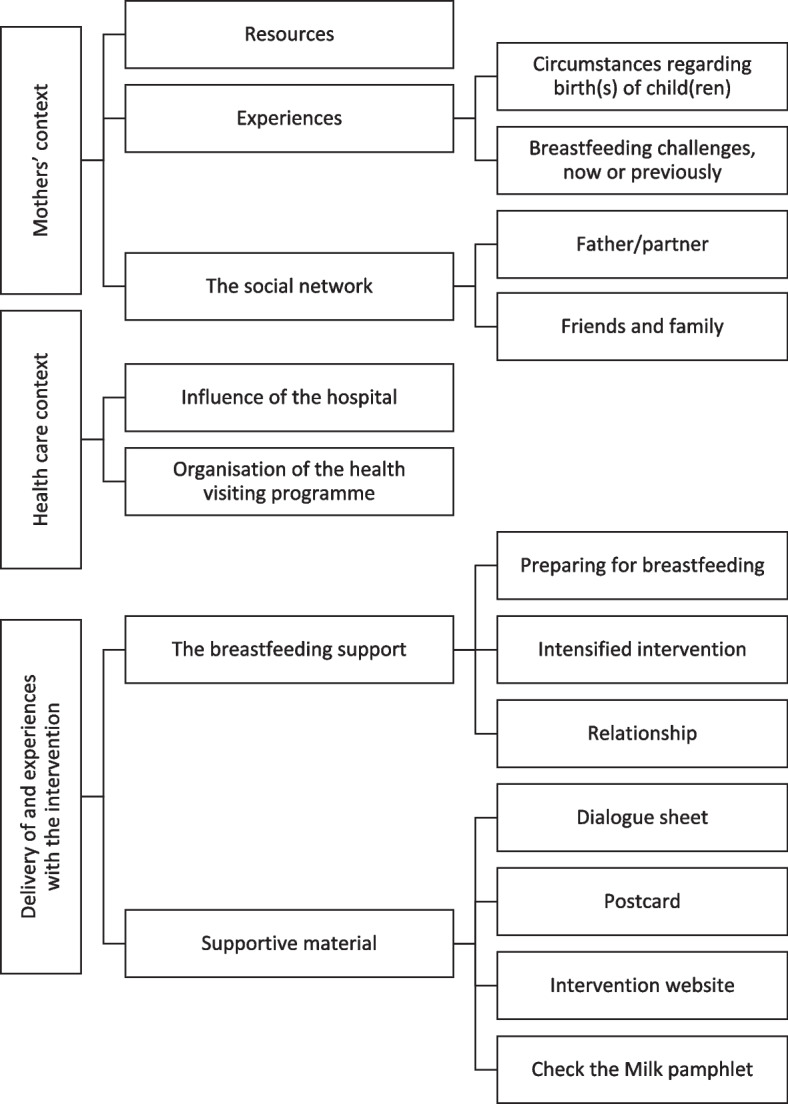
Coding tree – interviews with mothers

In the interviews with mothers, eight mothers and one father participated. Five mothers were eligible for intensified intervention, and four had accepted the offer. Mothers’ ages ranged from 21–32, and the infants’ age at interview ranged from < 1–9 months (median 4 months). Four had either primary school or vocational education, one had secondary education and three had tertiary education as their highest educational attainment. All participants were in a cohabitating relationship or married, three were primiparous and five multiparous. Five interviews were conducted face-to-face and three via telephone. A coding tree of the analysis of interviews can be found in Fig. 5.

### Implementation

#### Implementation process

Of the 134 health visitors in the intervention arm responding at follow-up, 90% (*n* = 120) responded to have participated in full, while two (1%) had participated in parts of the breastfeeding support education programme (data not shown). Twelve (9%) had not participated at all. Reasons for not participating in the education were ‘*not employed at the time*’ (*n* = 7) and ‘*leave*’ (*n* = 5) (data not shown). Of the 12 non-participants, 11 reported having received one-to-one education from a colleague at the least. The remaining health visitor had not received any education (data not shown).

Health visitors in focus groups expressed that the intervention fitted well within their usual practice and that the education programme had enabled them to apply in-depth knowledge about for example the physiology of milk production to their existing support.

“*I feel that the education programme confirmed much of my existing knowledge and also provided me with new insights. I found it useful, and I now apply the knowledge to tailor the information I provide to meet each family*’*s needs.”* (Health visitor 8)

#### Intervention delivery

**Fig. 6:**
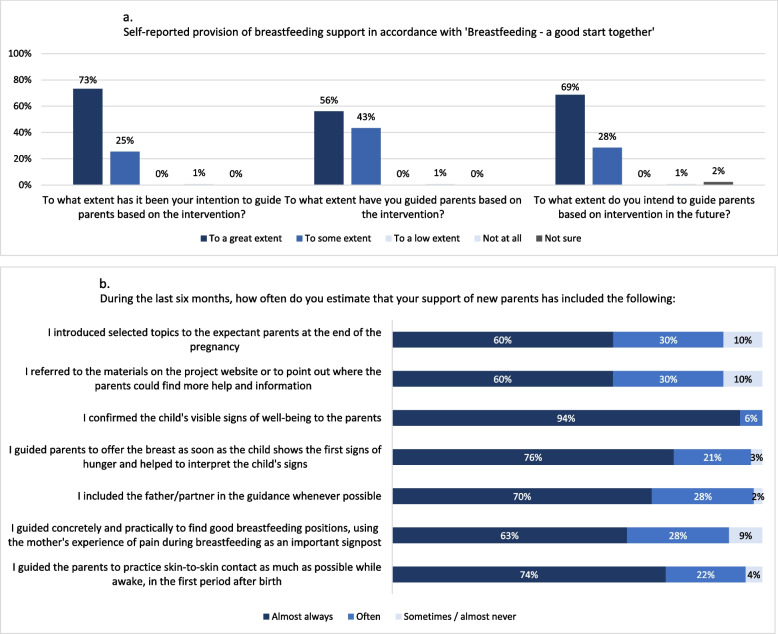
Health visitors’ self-reported delivery of the intervention breastfeeding support

Most health visitors in the intervention arm reported that they had intended to guide according to the intervention and had indeed done so. They also expressed their intention to continue guiding in this manner (Fig. 6a).

In Fig. 6b presents responses to questions regarding specific theory- and evidence-based elements of the intervention. Here, too, most health visitors (90–98%) reported that they ‘*often*’ or ‘*almost always’* delivered support in alignment with the intervention (Fig. 6b). This was confirmed by qualitative data, where health visitors described tailoring information to fit parents’ needs, and delivering the postcard, the “Check-the-Milk” pamphlet and the project website to all families they encountered. Mothers participating in the interviews confirmed this.

*“I think that the simpler it is with such a card (the postcard), the easier it is for them to comprehend. So, when you ask if the support we provide is the same as usual, yes, to some extent it is. But the postcard is a good tool because it adds something illustrative, which I think was lacking before. It helps underline the importance of the key messages.”* (Health visitor 2)

The dialogue sheet was an exception to consistent delivery. Health visitors met it with resistance and found it difficult to use, especially during the first chaotic visit following birth when they had many tasks to complete. Consequently, it was decided to use it only during pregnancy visits. However, its delivery remained incomplete, with most focus group participants believing it duplicated the recording of families’ individual case histories in the electronic record system, which they already did during pregnancy visits. They felt micromanaged to perform a task already covered by their usual work and therefore did not use it. A few health visitors disagreed, finding the dialogue sheet useful and believing it facilitated a deeper understanding of the families’ situations. Interview findings further confirmed the inconsistent use, as only two mothers had encountered the sheet. These two mothers found it beneficial to the conversation.

In focus groups, health visitors expressed that they included more facts about the physiology of breast milk production in their support. Moreover, some health visitors said to have focussed more on laid-back breastfeeding positioning and on the skin-to-skin approach when supporting families than previously because they had been reminded of these techniques in the education programme. Using the skin-to-skin approach to solve early breastfeeding problems was advised and had been successfully applied by a couple of the interviewed mothers. On the contrary, some of the mothers in the interviews exposed examples of the delivered breastfeeding support conflicting with the intended intervention. For instance, a few mothers had been guided to try to space out feedings to prevent an infant’s rapid weight gain, and another had been told that it was normal to experience pain related to breastfeeding in the beginning.

*“When I asked my health visitor, she said it was normal for breastfeeding to hurt in the beginning, because my body wasn’t used to it and such. But that it at least should get better. It did when I was almost finished breastfeeding. But eventually, it ran out (the milk). […].”* (Mother 3)

In a couple of the interviews with mothers, expressions revealed that the intervention element intended to retrain previous negative experiences might not have been fully implemented and consistently delivered.

*”So, I’ve basically given up. I thought to myself that this third time, I don’t want to try – the burden is too big.”* (Mother 8)

Combined, the empirical data implied that the communicative parts of the intervention, i.e., the dialogue sheet to ensure individualised breastfeeding support and enhancement of self-efficacy, the retraining of negative previous experiences and the evidence-based breastfeeding support messages, were inconsistently delivered.

Managers in the organisational survey reported that the implementation of the postponed four-month visit was successful, although sometimes difficult to implement due to barriers related to families’ context to be elaborated below (data not shown).

Mothers in interviews who were in the target group for the intensified intervention confirmed having received the planned intervention. However, focus groups revealed that not all target group mothers were offered proactive calls. Health visitors sometimes prioritised other pressing family circumstances over offering the intensified intervention or anticipated that certain families lacked the resources to focus on breastfeeding. In all three focus groups, a general perception conflicted with the rationale for offering the intensified intervention to young mothers and/or those with low educational attainment. Health visitors believed these mothers were generally better ‘breastfeeders’ because they did not overthink breastfeeding. Conversely, they found mothers with high educational attainment required more reassurance about their infants thriving on unmeasurable milk amounts. Thus, health visitors did not always agree that the target group mothers needed extra follow-ups. Consequently, the combined analyses indicate that health professionals’ judgement took precedence over allowing target group mothers to make an informed decision.

##### Dose

In intervention clusters that had not previously had pregnancy visits as part of their usual care, local representatives in dialogue meetings and health visitors in focus groups expressed a demand for pregnancy visits, making them easy to implement. Health visitor records showed an increase in delivery of pregnancy visits in most intervention clusters (Table 2), thereby confirming the ease of implementation. However, increases were also observed in most control clusters (Table 2) which might indicate some level of contamination across trial arms.

**Table 2:** Mean contacts per birth before and after implementation of the intervention across trial arms

	Pregnancy visits		Number of visits		Needs-based visits		Telephone contacts	
Intervention clusters	Before	After	Before	After	Before	After	Before	After
Cluster 1	0.4	0.5 ^↑^	6.5	7.5^↑^	1.9	2.5 ^↑^	2.5	3.1 ^↑^
Cluster 2	n/a	n/a	15.1^a^	19.5^a↑^	–	–	1.4	1.7 ^↑^
Cluster 3	0.6	1.1 ^↑^	5.6	6.9 ^↑^	1.1	1.2 ^↑^	n/a	0.5 ^↑^
Cluster 4	0.4	0.3 ^↓^	6.0	6.2 ^↑^	1.2	1.5 ^↑^	1.5	1.8 ^↑^
Cluster 5	0.2	0.3 ^↑^	5.3	5.7 ^↑^	n/a	n/a	1.1	0.9 ^↓^
Cluster 6	0.3	0.6^↑^	5.1	5.4 ^↑^	1.4	1.2 ^↓^	3.0	3.7 ^↑^
Cluster 7	0.5	0.9^↑^	6.0	7.0^↑^	1.5	1.9 ^↑^	3.0	4.6 ^↑^
Cluster 8	0.5	0.6^↑^	8.9	7.7 ^↓^	1.3	1.0 ^↓^	3.8	3.8^→^
Cluster 9	1.0	1.8^↑^	5.5	6.5 ^↑^	1.4	1.6 ^↑^	2.9	4.1 ^↑^
Cluster 10	1.1	1.1^→^	7.1	6.3 ^↓^	2.3	1.6 ^↓^	5.3	4.9 ^↓^
Control clusters	Before	After	Before	After	Before	After	Before	After
Cluster 11	0.8	0.8^→^	8.0	6.6 ^↓^	2.0	1.6 ^↓^	3.2	2.4 ^↓^
Cluster 12	0.2	0.3 ^↑^	6.7	6.8 ^↑^	1.4	1.4^→^	2.4	2.7 ^↑^
Cluster 13	0.4	0.5 ^↑^	6.4	7.3 ^↑^	1.4	1.6 ^↑^	2.2	2.0 ^↓^
Cluster 14	0.7	0.9 ^↑^	5.7	6.3 ^↑^	1.6	2.0 ^↑^	2.3	2.4 ^↑^
Cluster 15	0.3	0.4 ^↑^	6.5	6.1 ^↓^	2.0	1.6 ^↓^	1.1	1.3 ^↑^
Cluster 16	0.7	0.9 ^↑^	5.6	5.8 ^↑^	1.4	1.5 ^↑^	4.1	3.9 ^↓^
Cluster 17	0.6	0.9 ^↑^	6.4	6.2 ^↓^	3.5	3.3 ^↓^	2.1	2.0 ^↓^
Cluster 18	0.6	0.6^→^	6.2	6.9 ^↑^	1.4	1.2 ^↓^	2.5	2.3 ^↓^
Cluster 19	0.5	0.5^→^	6.9	7.0 ^↑^	2.4	1.7 ^↓^	3.1	3.6 ^↑^
Cluster 20	0.6	0.7 ^↑^	4.9	5.3 ^↑^	1.4	1.6 ^↑^	0.5	0.4 ^↓^

Periods: *Before* = October 2021 – March 2022; *After* = August 2022 – July 2023; *Intermediate* = April 2022 – July 2022. The intermediate period is not reported to allow for an implementation period in the intervention clusters. The intervention clusters underwent education during March 2022 and began implementation the with implementation following completion of the education

Arrow pointing down indicates a drop, arrow pointing right indicates no change, while arrow pointing down indicates an increase in mean contact over time periods

Source: Health visitor records

^a^The specific electronic record system did not support extraction based on infants aged 0–6 months. Thus, numbers reported are higher than the remaining municipalities because they include contacts regarding children above 6 months of age

The health visitor records were ambiguous regarding telephone contacts but tended to converge with the data from the interviewed mothers in the target group, who confirmed having received the planned calls. The mean number of telephone calls per mother-infant dyad increased in seven out of the 10 intervention clusters after the implementation of the intervention (Table 2). This increase was also noted in some control clusters, although in less than half of the clusters.

##### Reach

The reach of the intensified intervention was compromised, as some health visitors in focus groups found it difficult to explain the motive for offering it to mothers in the target group without stigmatising them. Others found it easy, suggesting that finding a comfortable way to voice the offer was key. As unfolded above, additionally, health visitors noted that in some situations, more pressing circumstances took priority over offering the intensified intervention.

*”I think it depends on how well you ’sell it’ (the intensified intervention), and I think that some might consider, in this particular family, there is no point in even introducing it. At least in our team, when we discussed it yesterday, some of my colleagues had no families in the intensified intervention, because they had reasoned, ‘They are not able to… It will be too much’. And so, the families may not even get the offer”.* (Health visitor 1)

##### Adaptation

As mentioned, the dialogue sheet was adapted for use during pregnancy visits only, but this had little impact. Qualitative data indicated that the intensified intervention also underwent significant adaptations in terms of delivery mode and schedule. Health visitors wished to include text messages as a delivery mode. The interviewed mothers confirmed this by having received text messages. Additionally, some health visitors autonomously adjusted the contact schedule to fit their individual timetables. For instance, planned holidays could disrupt the schedule, leading some health visitors to make the contact before or after the planned time to avoid having colleagues take over, while others left it to a colleague to handle.

### Mechanisms of impact

#### Participant responses to and interaction with the intervention

**Fig. 7:**
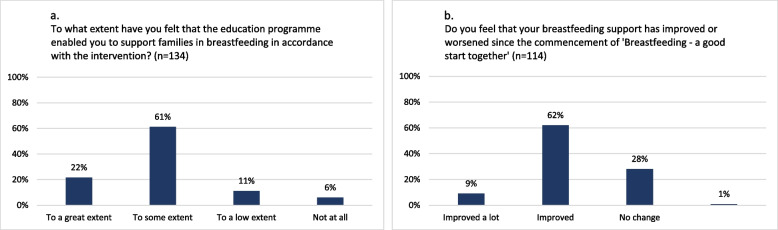
Health visitors’ self-reported assessment of the education programme and their breastfeeding support

Of the respondents in the intervention arm, most (83%) felt that the education had enabled them to deliver the intervention breastfeeding support (Fig. 7a).

The health visitors reported having intended to and intending to continue to deliver breastfeeding support in alignment with the intervention (Fig. 7a), underlining their acceptance of the intervention.

*“We really want to support breastfeeding, so if there is anything we can do to increase the breastfeeding rates then that’s valuable. That’s what motivate us […]”* (Health visitor 1)

In focus groups, they noted that the intervention breastfeeding support was like their usual support but simpler and clearer. The four key messages structured the support and helped them remember the most important things.

**Fig. 8:**
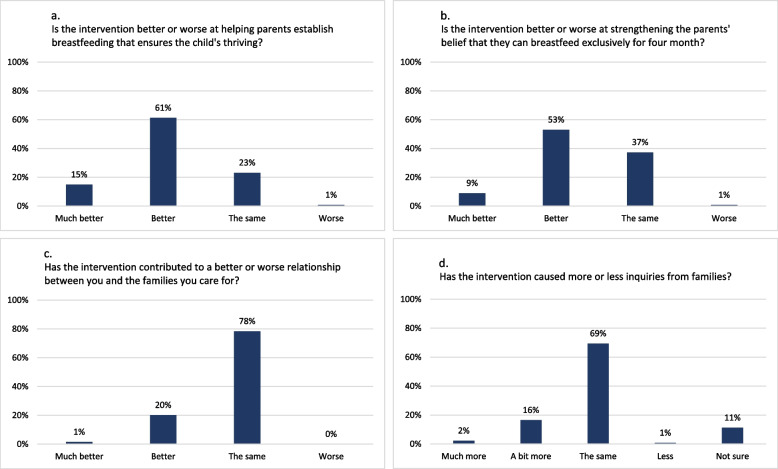
Health visitors’ self-reported comparison of the intervention breastfeeding support to usual breastfeeding support (*n*=134)

Most (71%) health visitors in intervention clusters with complete responses felt that their breastfeeding support had improved since the implementation of the intervention (Fig. 7b). They believed the intervention was superior to usual breastfeeding support in establishing breastfeeding for infant thriving (Fig. 8a) and in strengthening parents’ confidence to breastfeed exclusively for four months (Fig. 8b). However, regarding relationship formation with families and number of inquiries from families since the intervention commenced, health visitors largely reported the intervention to produce similar effects to usual support (Fig. 8c-d).

**Fig. 9:**
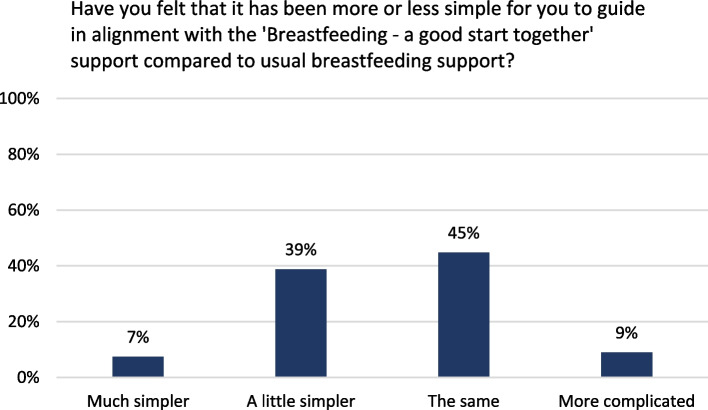
Health visitors’ self-reported assessment of the intervention breastfeeding support (*n*=134)

All health visitors in focus groups appreciated the postcard, the Check-the-Milk pamphlet, and the website, finding them appealing and helpful for providing support via telephone. They noted that the postcard with the four key messages structured and simplified breastfeeding support by putting focus on the most important aspects. Most health visitors in the survey confirmed this, reporting that providing breastfeeding support in alignment with the intervention was simpler or the same as before (Fig. 9).

**Fig. 10:**
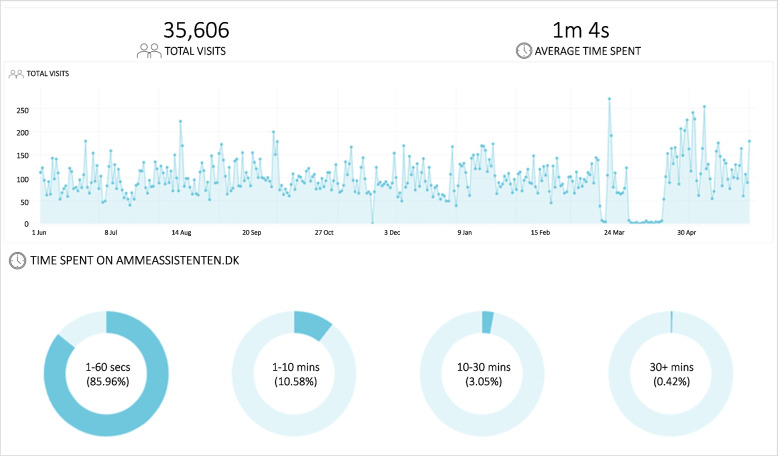
Total visits* and time spent per visit on the intervention website June 2022-June 2023. *Around the beginning of April there was unfortunately an error on the website, which causes the graph to drop to zero in a period of about a fortnight until it was corrected

Health visitors in focus groups and dialogue meetings found the intervention website useful and noted that there had been a demand for sound, evidence-based information about breastfeeding in Danish. They were surprised that families seemed not to have utilised the website much. Website data, however, showed over 35,000 visits in a one-year period, with most visits (86%) lasting less than a minute, 11% lasting between one to 10 min, and the remaining 3% lasting 10–30 min or more (Fig. 10). Despite most visits being short, the visits lasting more than 10 min amounted to over 5,000 during the year, suggesting that families used the website when they felt the need.

This varied use of the site was supported by interview data. Most mothers expressed having taken a quick look, while a few spent a lot of time on the website. Notably, mothers found the many videos on the website offered timely and useful support. Watching videos was easier than reading while holding an infant, although some mothers and their partners also found other materials, such as texts and podcasts, useful.

*“Yeah, so if there was something we doubted, then he (the father) was the one reading about it and then passing on the information. So, he has used it (the website).”* (Mother 6)

Website data supported this preference for videos, with the ten most visited pages all being videos (Table 3). Health visitors expressed a desire for continued use of the project website and postcard after the project ended. Interview data confirmed that mothers who had found the intervention website useful wished to have continued access and wanted others to benefit as well.

**Table 3:** Ten most popular webpages on the intervention website June 2022—June 2023

Webpage	Total visits in period
Breastfeeding positions (video)	32,830
Signs of thriving—the child's signs of hunger and satiety (video)	22,838
Laid-back breastfeeding (video)	11,322
Good suckling technique (video)	6,775
Skin-to-skin principle to start over again with breastfeeding (video)	6,681
Breastfeeding with nipple shield (video)	5,029
Preparation for breastfeeding (video)	3,671
Hand expression of breastmilk (video)	1,283
Breastfeeding during the first period (video)	884
How breastfeeding works (physiological explanation) (video)	676

Health visitors’ reactions to the intensified intervention ranged from viewing it as a ‘time-consuming waste’ to believing ‘it works really well’. Some believed that families in need would reach out on their own, making the proactive approach unnecessary. Others found the intervention effective, had experienced being included in discussions about discontinuing breastfeeding and sometimes able to postpone it. They had experienced preventing discontinuation in multiparous families who had previously started bottle-feeding at a certain point. Additionally, in families concerned about insufficient milk supply due to the emergence of more frequent feedings, health visitors were able to explain that infants increase milk production by breastfeeding more often, which likely caused the change in feeding pattern. At the very least, they felt the additional contact provided parents with reassurance and motivation to continue breastfeeding.

*“[…] I have been contacted by parents in the intensified intervention, ‘We just want to ask you what you think about this – would it be okay to bottle-feed tonight?’. […] I have been able to prolong the breastfeeding by informing them, ‘Well, a lot is happening with your baby emotionally and that’s why the baby wakes up and cries out for you more right now – it’s not just about the breastfeeding’. […] Or, ‘The baby eats faster now and gets more milk at a time, and that’s why the feedings are faster now.’”*

(Health visitor 11)

Data from interviewed mothers expanded focus group findings, revealing that frequent contacts from the health visitor might work by making mothers feel that reaching out would be no inconvenience. If the health visitor had time to call them frequently, they reasoned, she would likely also have time for the mother’s call or text message.

Health visitors believed that the proactive telephone calls could potentially save time by replacing some needs-based visits. They foresaw using these calls to assess the need for a visit instead of making the visit ‘just in case’. They agreed that while the intensified intervention could be beneficial for some families in the target group, the decision should be based on their professional judgement.

In focus groups, health visitors emphasised the importance of pregnancy visits in forming relationships with families and understanding their breastfeeding intentions before the infant’s arrival. This allowed them to tailor support based on previously expressed wishes and current needs.

*“I find it important when we are on pregnancy visits to ask them when I show them the postcard, ‘What do you think? Do you want to breastfeed? Did you talk about it?’ […]. So that it is a choice and not something that I tell them to do. I think it is important, because then you talk about their wishes and sense how strongly they feel about it. […]. It is so important that you kind of balance it out, ‘What was it that we talked about back then, when they didn’t have those sore breasts and cracks and bleeding and were tired’”* (Health visitor 1)

Interview data confirmed this, with one mother noting that the health visitor’s knowledge of her wishes before challenges arose led to timely encouragement to give it another go, consequently facilitating continued breastfeeding that would have otherwise been discontinued.

Regarding the postponement of the four-month visit, the organisational survey showed managers viewed the uptake positively and wished to maintain the practice post-intervention. Health visitors in focus groups found the postponement meaningful but had some concerns about potential adverse effects (reported below).

#### Unexpected pathways and consequences

During dialogue meetings and in focus groups, concerns were raised about the risk of stigmatising mothers in the target group for intensified intervention by voicing the eligibility criteria. Interview data refuted this, as none of the interviewed mothers felt stigmatised. Some mothers did not fully comprehend the motives for the offer. Confirming this, in a focus group a health visitor said that her manager advised the team to had omit the motive when offering the intervention as a help to overcome the barrier. Another concern raised in focus groups, was that the intensified intervention might inadvertently introduce insecurities if mothers perceived telephone calls as monitoring. Again, interview data disproved this, with mothers reporting that close follow-up actually fostered a sense of security.

*”Yeah, I thought it was super nice (receiving the proactive calls). I remember being a bit nervous (prior to the first visit) about, ‘Oh no, will I be judged for being… you know… young’. But she was super nice, and it was quite comforting to know, like… Because she ensures me that she is only trying to help me. […] It felt very safe for me […].”* (Mother 2)

As mentioned earlier, postponing the four-month visit raised concerns about delaying the detection of inadequate motor skills in infants. Health visitors in focus groups noted that postponing the visit could be challenging in practice. Some families, particularly the multiparous-, expected a four-month visit, while first-time parents were eager to start introducing solids into their infant’s diet.

### Context

A mother’s resources, such as psychosocial-, educational- and mental resources, facilitated the intervention by enabling her to set breastfeeding intentions and thereby receive partner support, cope with challenges, seek help from others when needed and critically appraise the advice and support received.

*” If he hadn’t pushed me to express my milk… Or not push, but support me, then I would have stopped (breastfeeding) entirely and gone with formula”* (Mother 2)

Overall, having resources helped a mother succeed in breastfeeding. However, the intervention was designed to enhance a mother’s self-efficacy, among other things by identifying individuals in her social network able to support with good breastfeeding accounts, and build action competence to overcome obstacles, by for example providing knowledge and evidence-based support. When mothers with fewer resources struggled to overcome challenges, it may indicate parts of the intervention not being delivered as intended.

Another contextual factor was parity. Parity could for instance hinder the postponement of the four-month visit. Managers of intervention municipalities confirmed that the families’ context could impede the postponement. Mothers in interviews expanded multiparity to facilitate intervention mechanisms when previous experiences provided the right prerequisites to prepare for breastfeeding and seek needed help, for example from the hospitals’ antenatal care and during pregnancy visits. Conversely, repeated negative experiences with breastfeeding could lead to giving up.

Similarly, members of ‘mothers’ groups’ could support each other in breastfeeding, or conversely praise the advantages of infant formula and thereby end up creating a ripple effect. As expressed by a health visitor:

“*I have seen the impact of mothers’ groups, especially when they discuss bottle feeding […]. When I visit, some mothers tell me, ‘We have started bottle feeding’, and I wasn’t involved. I ask, ‘Where did you get the idea?' and they say, ‘We heard from the mothers' group that it was really good’. So, I believe mothers' groups influence people’s choices. It depends on the group – whether everyone breastfeeds or a large proportion bottle feed. That’s another challenge we face.”* (Health visitor 7)

Additionally, the health care context impacted the intervention. Mothers generally found the breastfeeding support in hospitals consistent with that provided by the health visitors. However, they also recounted instances where the support counteracted the intervention, such as when health professionals in hospital handed out nipple shields to alleviate pain or instilled notions about the necessity of supplementing with formula until the milk had come in.

*” […] We were at the hospital on day two (after birth), and then a couple of nurses came in, and then she (the infant) was fed some formula, because (they said) it can take a while for your milk to come in properly.”* (Mother 1)

**Fig. 11:**
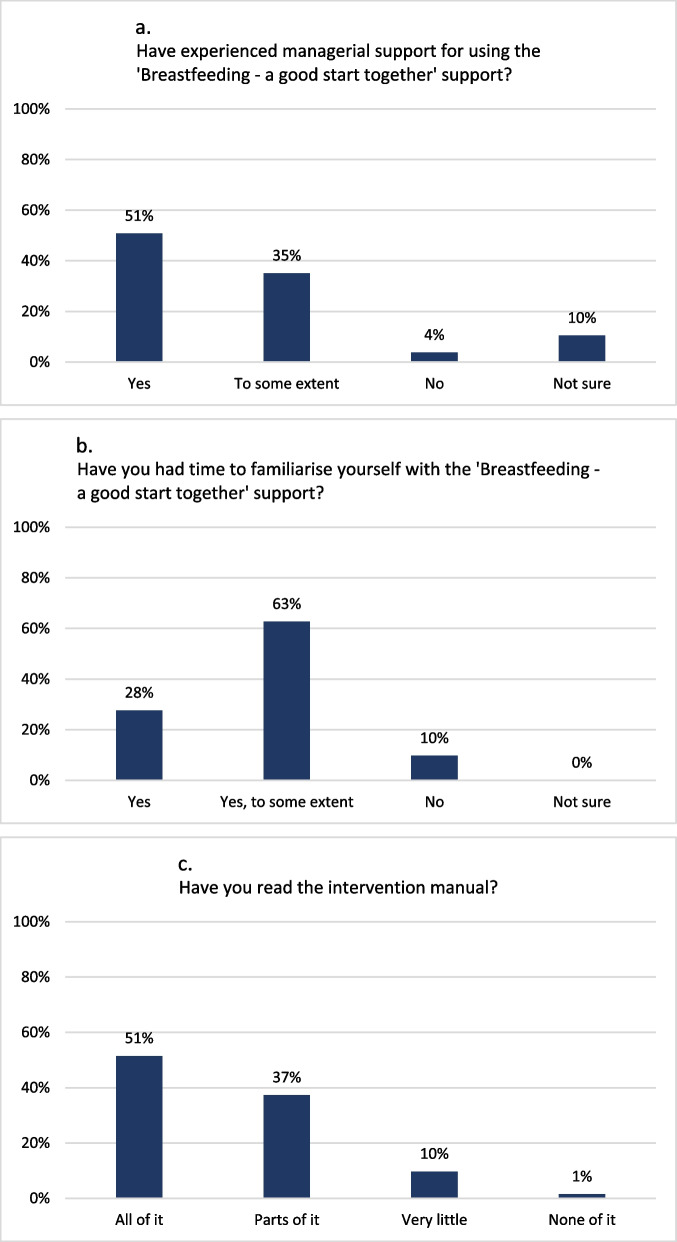
Health visitors’ self-reported implementation of the intervention breastfeeding support (*n*=134)

In focus groups, health visitors highlighted the importance of management for the implementation, for instance by recruitment tasks to fit the context or by prioritising resources to the project. Participation in the education programme fostered a sense of ownership in the health visitors. However, newly employed health visitors could end up impeding intervention implementation and delivery and some health visitors in focus groups noted variation in colleagues’ attitude towards the intervention. The health visitor survey showed that most participants had received managerial support (Fig. 11a), countering concerns that a lack of support and ownership might threaten delivery. Additionally, most health visitors reported having had time to familiarise themselves with the intervention and had read the manual (Fig. 11b-c), indicating that the organisations had allocated the necessary resources for implementation.

Two other organisational factors affecting implementation were workload and financial resources. In focus groups, health visitors noted that the intensified intervention’s workload was unevenly distributed within teams due to differences in the population composition in each health visitors’ assigned district. Organisational survey data showed that nearly twice as many clusters in the control arm (control: 7 vs. intervention: 4) had health visitors specifically appointed to care for particular groups, such as for instance young mothers or mothers of low educational attainment (Table 1). Financial cutbacks to the health visiting programme strained resources and individual health visitors, thereby impeding the implementation, as pointed out by focus group participants.

The health visitor survey revealed more frequent (informal) discussions about breastfeeding in the intervention arm than in the control arm (Table 4), complementing organisational survey findings on the frequency of team meetings (Table 1).

**Table 4:** Comparison of contextual elements reported by health visitors at follow-up. Both trial arms

	**Control group** *n* = 150	**Intervention group** *n* = 134	**Comparison**
	**n (%)**	**n (%)**	*p*-value*
**How often did you discuss questions regarding breastfeeding with your colleagues during the past month?**			0.009
Once or several times a week	60 (40)	64 (48)	
1–3 times during the last month	80 (54)	64 (48)	
I have not discussed questions about breastfeeding during the last month	9 (6)	6 (5)	
**Have you provided more or fewer needs-based visits than usual related to breastfeeding during the last six months?**			0.001
More	25 (17)	42 (31)	
The same	116 (77)	76 (57)	
Fewer	0 (0)	1 (1)	
Not sure	9 (6)	15 (11)	

^*^P-values calculated using Fisher’s exact test

In dialogue meetings, it became clear that resourceful local representatives in team meetings and discussions facilitated the implementation and delivery of the intervention by adapting it to fit practice. Health visitors in focus groups confirmed this, noting that local representatives further helped maintain focus on the intervention, provided pep talks when needed, and fostered a sense of ownership through direct links with intervention developers, allowing for quick resolution of questions and continuous adaptations.

“*Well, I think that the occasional peptalk... And that the distance (to the research team) isn’t that great. We’ve experienced changes, for example, ‘It doesn’t make sense to call this mother – it feels stupid, because she just breastfeeds […]’. So, she (the local representative) would ask if it’s okay to just send a text message. And that was fine. Yeah, it makes you feel like we have influence.”* (Health visitor 16)

Conversely, when local representatives did not provide pep talks or include the intervention in team meeting agendas, focus on the intervention diminished.

The intervention’s integration into a large trial was a frequent topic in dialogue meetings. The eligibility criteria for recruiting families to the trial, and specifically to the intensified intervention, placed a double burden on health visitors, who also had to deliver the intervention. Focus group findings confirmed this and further revealed that the project infrastructure acted as both a barrier and a facilitator. It caused confusion and frustration but also ensured focus on the intervention and helped health visitors remember to deliver intervention materials to families.

*“[…] I find that my focus sharpens when I sit with the family and must present the breastfeeding project. I introduce the postcard and the website, and we talk about enrolling them in the study and what it involves. Then we’re already caught up in talking about breastfeeding […]”* (Health visitor 13)

In both trial arms, there were shifts in management and staff turnover during the project period (Table 2). Although the differences between groups were not statistically significant, the intervention arm had visually higher proportions, with 50% of management and 18% of staff replaced since the project started, compared to 30% and 12% in the control arm.

### Intervention fidelity

Overall, the findings suggest that most intervention elements, including supportive materials, were delivered with high fidelity. However, the communicative aspects, such as the dialogue sheet for tailored support, communicative support to improve self-efficacy, and addressing negative breastfeeding experiences, were less consistently delivered with fidelity. Additionally, the intensified intervention was not consistently offered to the target group.

Health visitors reacted positively to the intervention, noting that it fit well into their usual practice, provided structure, simplified their breastfeeding support, and improved families’ chances of breastfeeding, thus facilitating implementation. Mothers in interviews expressed receiving the intervention as planned and reacted positively to both the intervention materials and the intensified support.

Contextual factors impacting the implementation and delivery included staff and management turnover, the project infrastructure that required health visitors to both deliver the intervention and recruit for the trial, and the mothers’ individual contexts influencing the mechanisms of impact.

## Discussion

The aims of the present study were to assess the implementation of the intervention, explore the intervention’s mechanisms of impact, identify contextual factors that might affect the intervention, and evaluate overall fidelity to the intended intervention. Conducting the process evaluation prior to analysing the effectiveness of it was important for the authors, to remain unbiased towards the processes [12].

We found most of the intervention elements to have been delivered with high fidelity, with some exceptions, and a general appreciation of the intervention across health visitors and the mothers receiving the intervention.

A variety of findings across our data will influence the interpretation of the future effectiveness study and decisions on potential scaling of the intervention. Some findings suggest that the intervention may be applied beyond the project clusters to the wider Danish health visiting programme.

Firstly, the content of the health visiting programmes across the trial arms, after the introduction of the intervention, was found to be largely similar. This indicates that parents in the control arm may have received support very similar in structure to the intervention. Despite this, due to the randomised nature of the study and statements from health visitors in the intervention arm about how their breastfeeding support had become simpler and more structured, we reason that the quality of support received across trial arms likely differed. Moreover, providing simpler breastfeeding support may be more comprehensible for mothers with varying levels of health literacy, potentially reducing social inequity.

Pregnancy visits were offered in most clusters in both trial arms, and a higher proportion of control clusters reported having appointed health visitors to specifically handle care for special groups, such as the target group for the intensified intervention. This suggests that scaling the intervention may be feasible in the Danish context. Transferability to other settings will need to consider the interaction and fit between the intervention and context [27].

Secondly, forming trustful relationships between mothers and health professionals, open communication about breastfeeding goals and continuity in support have been proven to facilitate tailored and effective breastfeeding support [28, 29]. This was confirmed by the mothers in this study. Qualitative studies of breastfeeding support for vulnerable groups have found that the social network is crucial in supporting lactating women, and some women choose not to consult health professionals despite having needs [6, 30]. Our results suggests that pregnancy visits help facilitate the intervention delivery by establishing a foundation for the relationship and preparing women for breastfeeding. Additionally, the intensified intervention seemed to encourage mothers to reach out to health visitors.

However, the inconsistent use of the dialogue sheet to facilitate relationship formation, enhance mothers’ self-efficacy and build action competence might have impeded the intervention’s mechanisms of impact. This is supported by interview data indicating that some communicative elements were not consistently delivered. Contextual factors such as staff turnover may have further affected continuity of care, though not to an extent than hindered the intervention mechanisms significantly. We believe that the intervention’s effectiveness on breastfeeding rates and duration is likely, despite the absence of the dialogue sheet element, because pregnancy visits helped health visitors provide tailored support. We recommend exploring the impact of proper breastfeeding preparation in pregnancy in other contexts.

Thirdly, fathers/partners have a significant influence on the breastfeeding, either by supporting or undermining it [31]. Our empirical data from interviews with mothers support this, and our analysis further expands it, showing that the mother’s expressed intention to breastfeed catalyses this influence. This finding supports the intervention element to provide breastfeeding support to both parents, allowing mothers to express their intentions and for partners to back them up. Future analyses in the Breastfeeding Trial will explore the impact of including fathers/partners in breastfeeding support, providing a deeper understanding of the intervention mechanisms.

Fourthly, health visitors in the intervention arm reported discussing breastfeeding with colleagues more frequently than those in the control arm, which may have facilitated implementation. According to Chesnel et al. [32], discussing support practices with colleagues is an important part of breastfeeding education. However, frequent discussions may impede the entire team’s support if the information is outdated. We recently established that the education programme was effective in improving health visitors’ self-reported knowledge, self-efficacy and action competence [9]. Health visitors in this study highlighted gaining knowledge as a major benefit of the intervention. Despite this, some mothers experienced support that contradicted the intervention education, indicating that changing practice and unlearning routines is challenging, as previously suggested by Dykes [33]. This suggests that the knowledge constructs investigated in the previous article may have overlooked important components or that the educational programme did not adequately address unlearning of outdated or wrong convictions as a prerequisite for new learning [34]. If health visitors retained flawed knowledge despite new training, this may explain the delivery of intervention-counteracting messages. Consequently, revisions of the intervention should ensure collegial discussions and include unlearning of outdated practices. Both factors are likely relevant across various contexts.

Finally, factors external also influenced the implementation and mechanisms of the intervention. During the intervention period, Denmark introduced earmarked paternity leave, allocating 11 weeks of the 52-weeks parental leave to the father/partner [35]. While we do not contest the political context for this legislation, it may have counteracted the intervention by accelerating the introduction of solid food into the infants’ diets to accommodate mothers returning to work, whereas the intervention intended to postpone this. Internationally, Danish parental leave, although reformed, ensures good circumstances for family wellbeing [36] and is among the most privileged globally. If future studies find that the intervention effect is overshadowed by the parental leave reform, the intervention might face even greater challenges in countries with less favourable leave policies.

Considering all the above, we still believe that the intervention has the potential to improve breastfeeding rates and reduce social inequity in Denmark. The approach is highly acceptable to healthcare providers, and families with lower health literacy levels may find four simple messages easier to understand than a range of recommendations. Adapting the intervention to other contexts will require consideration of the contextual factors impacting its mechanisms. However, simplifying breastfeeding support to focus on four evidence-based messages appears to be a promising approach that could be implemented and tested in other settings.

### Strengths and limitations

This process evaluation has many strengths, including the variety of data sources, systematic coverage, and analytical integration. Collecting data in the control clusters provided insight into the contextual factors across the trial arms. Additionally, we invited health visitors not already involved as local representatives to the focus groups, anticipating that local representatives would be more positive about the project and breastfeeding in general than their colleagues. Although selection bias was a risk, it seemed low as health visitors in the focus groups expressed diverse opinions about the project.

The study also has several limitations. Firstly, collecting data for the process evaluation before completing the data collection might have increased the focus on breastfeeding in the control clusters, potentially leading to contamination across trial arms. Secondly, the data themselves have limitations, such as self-report bias, the quality of data extracted from health visitor records, selection bias in both the health visitor survey and qualitative data collection, and the conduct of focus groups online. The moderator’s role in online focus groups is generally similar to regular focus groups but requires a more active role to maintain a steady flow of communication [37]. The online focus groups in this study bordered on structured group interviews. However, the trade-off was that all participants voiced their opinions, ensuring no participants generated data alone. Thirdly, due to the multitude of data sources, the analyses only scratched the surface of intervention mechanisms. Future planned studies using Realist Evaluation methods aim to explore specific mechanisms of change in the intervention in more depth [8]. Finally, the process evaluation may lead to potentially strengthened breastfeeding support due to the enhanced focus on the intervention. Therefore, even though further investigation of the support in the control arm could have illuminated similarities and differences across trial arms, we chose to interfere as little as possible in the control arm to reduce the risk of contamination [38].

## Conclusion

The overall fidelity of the intervention delivery was high, although some communicative elements of the intervention were not consistently delivered. Health visitors found the intervention to fit well within their practice, structuring and simplifying their breastfeeding support. Parents were positive about the support received and the intervention materials, and they found the proactive telephone calls in the intensified intervention provided a sense of security. Organisational factors such as staff and management turnover acted as barriers to implementation.

This study offers a lens through which to view the upcoming effectiveness evaluation. Interventions that enable healthcare providers to deliver simplified and structured breastfeeding support, like the one studied here, may increase breastfeeding rates and reduce social inequity in breastfeeding in various contexts, both nationally and internationally. This approach is highly acceptable to healthcare providers and potentially easier for families with lower health literacy levels to comprehend and apply.

## Supplementary Information

Additional file 1.

Additional file 2.

Additional file 3.

Additional file 4.

Additional file 5.

## Data Availability

No datasets were generated or analysed during the current study.

## Abbreviations

BMI: Body Mass Index
IBCLC: International Board Certified Lactation Consultant
MRC: Medical Research Counsil
