# Supplementary material for: Process evaluation of a breastfeeding support intervention to promote exclusive breastfeeding and reduce social inequity: a mixed-methods study in a cluster-randomised trial
Source: Int J Equity Health. 2024 Oct 8;23:204. doi: 10.1186/s12939-024-02295-0 (PMC11463148; doi:10.1186/s12939-024-02295-0)
Supplement: Supplementary file 1 — Additional file 1. [file 12939_2024_2295_MOESM1_ESM.docx]

Additional File 1 | Overview of data sources for the process evaluation and the research questions they inform

| **Data source** | **Process evaluation theme and the related key functions**  (Moore et al. 2015) | **Research questions answered by data** |
| --- | --- | --- |
| ORGANISATIONAL SURVEY | Implementation |  |
|  | Dose | - Did the families in the intervention clusters receive the postponed introduction to solid food |
|  | Context |  |
|  | Contextual factors that shape the implementation and how the intervention works | - Did the intervention clusters have equal opportunity to deliver breastfeeding support as the control clusters? - What were the most important structural, physical, cultural, social and individual barriers and facilitators for the intervention? - Did the intervention clusters have important contextual factors that could impede or facilitate the implementation or delivery of the intervention? - If so, was this the case for control clusters as well? |
| HEALTH VISITOR SURVEY | Implementation |  |
|  | Process | - Did health visitors in the intervention clusters participate in the education programme? |
|  | Dose | - What did the health visitors report having delivered? |
|  | Reach | - Did health visitors offer the intended intervention to families in each target group? If not, why? |
|  | Adaptation | - How did the intervention unfold in practice? |
|  | Mechanisms of impact |  |
|  | Reactions to and interactions with the intervention | - How did the health visitors respond to the intervention? |
|  | Unexpected pathways and consequences | - Did the intervention produce unanticipated effects or negative consequences? |
|  | Context |  |
|  | Contextual factors that shape the implementation and how the intervention works | - Did health visitors participate in the training? - Was there contamination due to flux of staff across trial arms? - Did health visitors have managerial support for delivering the intervention? - Did health visitors have time to familiarise themselves with the intervention? |
| HEALTH VISITOR RECORDS | Implementation |  |
|  | Dose | - How many visits and telephone calls families have received? - Did families in intervention clusters receive more visits and telephone calls than families in control clusters? |
| WEBSITE DATA | Mechanisms of impact |  |
|  | Reactions to and interactions with the intervention | - How did the families respond to the intervention? |
| DIALOGUE MEETINGS | Implementation |  |
|  | Dose | - Were there implementation issues regarding delivery of certain elements? |
|  | Adaptation | - What adaptations have been made to the intervention? And why? |
|  | Mechanisms of impact |  |
|  | Reactions to and interactions with the intervention | - How did the health visitors and the families respond to the intervention? |
|  | Unexpected pathways and consequences | - Did the intervention produce unanticipated effects or negative consequences? |
|  | Context |  |
|  | Contextual factors that shape the implementation and how the intervention works | - What were the most important cultural, social and individual barriers and facilitators for the intervention? - What contextual factors affected (or was affected by) implementation, intervention mechanisms and outcomes? |
| FOCUS GROUPS WITH HEALTH VISITORS | Implementation |  |
|  | Dose | - What did the health visitors express having delivered? |
|  | Reach | - Did health visitors offer the intended intervention to families in each target group? If not, why? |
|  | Adaptation | - How did the intervention unfold in practice? |
|  | Mechanisms of impact |  |
|  | Reactions to and interactions with the intervention | - How did the health visitors and the families respond to the intervention? |
|  | Mediators | - Did the intervention work as planned? - Were the planned mechanisms of impact activated or did unforeseen mechanisms of impact occur? |
|  | Unexpected pathways and consequences | - Did the intervention produce unanticipated effects or negative consequences? |
|  | Context |  |
|  | Contextual factors that shape the implementation and how the intervention works | - What were the most important structural, physical, cultural, social and individual barriers and facilitators for the intervention? - What contextual factors affected (or was affected by) implementation, intervention mechanisms and outcomes? - Was there causal mechanisms present in the context that acted to sustain the status quo or potentiate effects? |
| INTERVIEWS WITH FAMILIES | Implementation |  |
|  | Dose | - What did the families report having received? |
|  | Reach | - Did families in the target group for the intensified intervention receive the proactive telephone calls? If not, why? |
|  | Mechanisms of impact |  |
|  | Reactions to and interactions with the intervention | - How did the end-users (families) respond to the intervention? - Which impact did the intervention seem to have? |
|  | Mediators | - Did the intervention work as planned? - Were the planned mechanisms of impact activated or did unforeseen mechanisms of impact occur? |
|  | Unexpected pathways and consequences | - Did the intervention produce unanticipated effects or negative consequences? |
|  | Context |  |
|  | Contextual factors that shape the implementation and how the intervention works | - What were the most important cultural, social and individual barriers and facilitators for the intervention? - What contextual factors affected (or was affected by) intervention mechanisms and outcomes? - Were there causal mechanisms present in the context that acted to sustain the status quo or potentiate effects? |
