# Supplementary material for: Process evaluation of a breastfeeding support intervention to promote exclusive breastfeeding and reduce social inequity: a mixed-methods study in a cluster-randomised trial
Source: Int J Equity Health. 2024 Oct 8;23:204. doi: 10.1186/s12939-024-02295-0 (PMC11463148; doi:10.1186/s12939-024-02295-0)
Supplement: Supplementary file 2 — Additional file 2. [file 12939_2024_2295_MOESM2_ESM.docx]

## Additional File 1 | Questions included in the organisational survey

| **Question** | **Derivative questions** | **Control** | **Intervention** |
| --- | --- | --- | --- |
| *Questions related to the management and size of the health visiting programme* | | | |
| Have you been a manager in the municipality since the beginning of the project in 2021? |  | X | X |
| No 🡪 | Where were you employed before? | X | X |
| Yes 🡪 | Did you take part in the decision about partaking in the research project? | X | X |
| Commentary box (optional) |  | X | X |
| How many newborns did you have in your municipality in 2021? |  | X | X |
| How many newborns did you have in your municipality in 2022? |  | X | X |
| How many IBCLCs do you have in your municipality? |  | X | X |
| How many health visitors (infant-focused) are employed in your municipality? |  | X | X |
| Commentary box (optional) |  | X | X |
| Have you had any shortage of health visitor staff in your municipality throughout the last year? |  | X | X |
| How many health visitors were hired during 2022? |  | X | X |
| If >0 🡪 | Of these, how many had just finished their education as a health visitor? | X | X |
| Commentary box (optional) |  | X | X |
| If control cluster 🡪 | Did you hire health visitors from one of the intervention clusters in the project? (listing of the municipalities encompassed) | X | X |
| Commentary box (optional) |  | X | X |
| *Questions regarding the organisation of visits in the municipality’s health visiting programme* | | | |
| Please mark all the visits you offer primiparous families:   - Pregnancy visit - Visit during the first week postpartum - Visit during the second week postpartum - Visit during first month postpartum - Visit when the infant is two months old - Visit when the infant is four months old - Telephone call when the infant is four months old - Visit when the infant is five-six months old - Telephone call when the infant is five-six months old - Visit when the infant is eight-ten months old - Other? Please indicate |  | X | X |
| Marked pregnancy visit 🡪 | How much time is allocated to pregnancy visits in your municipality? | X | X |
| Marked visit during the first week postpartum 🡪 | How much time is allocated to visits during the first week postpartum in your municipality? | X | X |
| Please mark all the visits you offer multiparous families:   - Pregnancy visit - Visit during the first week postpartum - Visit during the second week postpartum - Visit during first month postpartum - Visit when the infant is two months old - Visit when the infant is four months old - Telephone call when the infant is four months old - Visit when the infant is five-six months old - Telephone call when the infant is five-six months old - Visit when the infant is eight-ten months old - Other? Please indicate |  | X | X |
| Marked pregnancy visit 🡪 | How much time is allocated to pregnancy visits in your municipality? | X | X |
| Marked visit during the first week postpartum 🡪 | How much time is allocated to visits during the first week postpartum in your municipality? | X | X |
| Commentary box (optional) |  | X | X |
| Part of the intervention was designed to postpone the introduction of solid foods into the infants’ diet as far towards six months postpartum as possible. The need for a visit to be introduced to solid foods was to be assessed via telephone around four months postpartum.  What is your impression – have your municipality been successful in doing so?  Were there resistance/barriers? If so, which? |  | / | X |
| Are families in your municipality assigned a specific health visitor to follow them throughout their contact with the health-visiting programme? |  | X | X |
| Commentary box (optional) |  | X | X |
| Where do your planned visits under the health-visiting programme take place? |  | X | X |
| If outside the families’ homes 🡪 | Please expand on the organisation of the visits | X | X |
| *Estimations about compositions of family profiles in the municipality* | | | |
| What proportion of families in your municipality need one or more needs-based visits, would you estimate? |  | X | X |
| What is the most frequent cause that triggers needs-based visits? |  | X | X |
| What proportion of families in your municipality have declined the health-visiting programme in 2022, would you estimate? |  | X | X |
| Commentary box (optional) |  | X | X |
| *Organisation of staff, collegial sparring in the municipality, and conditions possibly impacting staff resources* | | | |
| How often do you have joint meetings with all infant focused health visitors in your municipality? |  | X | X |
| Do you have one or more health visitor(s) allocated to take care of specific groups of families, for instance young mothers, mothers with low educational attainment, or others? |  | X | X |
| Do you have a specific visiting profile or offer for vulnerable groups? |  | X | X |
| Commentary box (optional) |  | X | X |
| If your municipality participates in more research projects or projects that take up some of your resources, please elaborate here |  | X | X |
| Have other conditions in your municipality had an impact on your chance to implement the intervention “Breastfeeding – a good start together”? |  | X | X |
