# Supplementary material for: Process evaluation of a breastfeeding support intervention to promote exclusive breastfeeding and reduce social inequity: a mixed-methods study in a cluster-randomised trial
Source: Int J Equity Health. 2024 Oct 8;23:204. doi: 10.1186/s12939-024-02295-0 (PMC11463148; doi:10.1186/s12939-024-02295-0)
Supplement: Supplementary file 3 — Additional file 3. [file 12939_2024_2295_MOESM3_ESM.docx]

## Additional File 2 | Questions included in the health visitor survey

| **Questions** | **Derivative questions** | **Baseline** | | **Follow-up** | |
| --- | --- | --- | --- | --- | --- |
|  |  | **Control** | **Intervention** | **Control** | **Intervention** |
| *Questions about experience and educational background* | | | | | |
| Do you hold an education as health visitor? |  | X | X | X | X |
| Yes 🡪 | When did you finish your education? | X | X | X | X |
| Do you hold a certification as international lactation consultant (IBCLC)? |  | X | X | X | X |
| Yes 🡪 | When were you first certified? | X | X | X | X |
| Have you since the baseline questionnaire in December 2021 been under training to or received certification as international lactation consultant (IBCLC)? |  | / | / | X | X |
| What is your employment title? |  | X | X | X | X |
| *Questions about breastfeeding support* | | | | | |
| In comparison with your other duties in ordinary families with infants, how important do you think breastfeeding support is? |  | X | X | X | X |
| How certain are you that your breastfeeding support can:   - Help mothers with difficulty breastfeeding to establish an effective breastfeeding - Help the partner so that they can support the breastfeeding in practice - Strengthen the parents’ belief that they can establish an effective breastfeeding - Help parents using ‘hands off technique’ to get the infant to latch onto the breast, if it has difficulties latching - Help the mother using ‘hands on technique’ to get the infant to latch onto the breast, if it has difficulties latching - Help mothers who experience pain when breastfeeding, so that is does not hurt when the infant suckles - Increase parents’ ability to assess when the infant gets enough milk in the establishing phase - Increase parents’ ability to identify when the infant is hungry - Strengthen the parents’ belief that the infant gets enough milk in the establishing phase - Strengthen the parents’ belief that the infant gets enough milk beyond the first month of life - Help the parents prioritise skin-to-skin contact with their infant as much as possible during the first week after birth |  | X | X | X | X |
| To what extent do you feel capable of:   - Tailoring my breastfeeding support to match the parents’ needs - Using concrete situations in the breastfeeding support for active learning for the parents - Tailoring information to what the parents express a need to know - Offering practical knowledge (application knowledge) on how to breastfeed - Mapping possible breastfeeding problems - Find ways to deal with problems in close collaboration with parents - Setting realistic goals for action in the event of breastfeeding problems - Guiding and supporting parents in reaching the set goals - Involving the partner in the support on an equal basis with the mother |  | X | X | X | X |
| Do you feel that you have enough knowledge about breastfeeding in your day-to-day work? |  | X | X | X | X |
| If a healthy, mature infant during the first week after birth is sleeping and has not eaten for four hours, what will you advise the parents? |  | X | X | X | X |
| What do you think should be included in the breastfeeding support concerning when a mother should offer her healthy, mature infant the breast during the first days after birth? |  | / | / | X | X |
| How do you best assess if the infant gets the nourishment it needs when it is around one week old? |  | X | X | X | X |
| Indicate whether you agree or disagree with the following statements:   - During the first month of life the infant should be breastfed at least 8 times a day - If the infant has a pacifier during the early post-partum period, it can have problems latching onto the breast - If the infant suckles for too long in the beginning, the nipples will be damaged - Skin-to-skin contact between the infant and the mother’s partner can help the infant retain a good body temperature - If the infant’s breath smells of acetone during the first couple of days, it is a sign that it needs supplements of infant formula after breastfeeding - The milk production is regulated by the milk stimulating hormone Prolactin - If the infant is fed infant formula, the physiological transitioning after birth is affected - Infants that are nurtured by infant formula will get jaundice more often than infants nurtured by breast milk - Women that have a high educational attainment does not breastfeed for as long as women that have a low educational attainment - When the breast is full of milk, the milk production is inhibited |  | X | X | X | X |
| To what extent is it your intention to provide breastfeeding support in the following ways:   - To acknowledge the mother’s breastfeeding efforts in my support - To guide parents step-by-step on how they can breastfeed effectively - To identify the parents’ needs and tailor my breastfeeding support to them - To show parents (for instance via video, pictures, a doll, or by using myself) how they can breastfeed - To let parents try breastfeeding themselves and ask if they are unsure - To inform parents about factual knowledge solely based on their individual situation - Solely let the wishes and needs of the parents be the governing factor when breastfeeding problems need to be handled - To let the mother and partner (or another important person for the mother) participate equally when I provide support |  | X | X |  |  |
| How often do you experience that families spontaneously contact you with questions about breastfeeding outside the scheduled visits? |  | X | X |  |  |
| How often do you find that families spontaneously contact you if they are considering discontinuing breastfeeding? |  | X | X |  |  |
| How often do you estimate that your support of expectant or new parents during the last six months has included the following:   - I guided parents to practice skin-to-skin contact as much as possible while they were awake in the first period after birth - I guided concretely and practically to find good breastfeeding positions, using the mother’s experience of pain during breastfeeding as an important benchmark - I included the father/partner in the guidance whenever possible - I guided parents in offering the infant the breast whenever the first signs of hunger appeared and helped them interpret the infant’s signs of hunger - I confirmed the infant’s visible signs of thriving to the parents - I referred to the materials on the website “ammeassistenten.dk” to guide parents to a place where they could get more help and information ^a^ - I introduced chosen topics for expecting parents in late pregnancy |  |  |  | X | X |
| Have you had additional or fewer needs-based visits than usual regarding breastfeeding during the last six months? |  |  |  | X | X |
| How often during the last month have you discussed questions related to breastfeeding with colleagues? |  |  |  | X | X |
| To what extent do you feel that you and your colleagues provide breastfeeding support uniformly? |  |  |  | X | X |
| Commentary box (optional) |  |  |  | X | X |
| *Questions about the intervention, the training programme, and the intervention material* | | | | | |
| Did you participate in the training programme for health visitors that is related to the project ‘Breastfeeding – a good start together’? |  |  |  |  | X |
| Yes 🡪 | How much of the training did you participate in? |  |  |  | X |
| No 🡪 | What was the reason that you did not participate? |  |  |  | X |
| I was not employed by the time of the training programme 🡪 | When were you employed? |  |  |  | X |
|  | Did you participate in the training programme for health visitors that is related to the project ‘Breastfeeding – a good start together’ (March 2022) in a previous employment? |  |  | X^b^ | X |
|  | Did you receive training in the breastfeeding support provided in ‘Breastfeeding – a good start together’ by your colleagues? |  |  |  | X |
|  | Please describe the training you received (for instance, completed the e-learning, collegial training, read manual, other) |  |  |  | X |
| Compared to usual breastfeeding support, do you think that the support in ‘Breastfeeding – a good start together’ is better or worse in helping parents establish a breastfeeding that ensures the thriving of the infant? |  |  |  |  | X |
| Compared to usual breastfeeding support, do you think that the support in ‘Breastfeeding – a good start together’ is better or worse in helping parents believe that they can achieve exclusive breastfeeding for four months? |  |  |  |  | X |
| Compared to usual breastfeeding support, do you think that the breastfeeding support you provide has become better or worse since the implementation of ‘Breastfeeding – a good start together’? |  |  |  |  | X |
| Compared to usual breastfeeding support, do you feel that providing support in line with ‘Breastfeeding – a good start together’ is simpler or more complicated? |  |  |  |  | X |
| Compared to usual breastfeeding support, do you feel that the support in ‘Breastfeeding – a good start together’ has given you better or worse relationships with the families you support? |  |  |  |  | X |
| During the ‘Breastfeeding – a good start together’ project, did you experience that it was easy or difficult to:   - Use the parents’ perspective as a starting point - Use specific situations for active learning - Set realistic goals for action with the parents in case of breastfeeding problems, and guide and support them in achieving the goals |  |  |  |  | X |
| Consider your breastfeeding support during the ‘Breastfeeding – a good start together’ project period:   - To what extent was it your intention to support parents in line with the intervention? - To what extent did you support parents in line with the intervention? - To what extent is it your intention to continue supporting parents in line with the intervention in the future? |  |  |  |  | X |
| In your estimation, have you received additional or fewer inquiries related to breastfeeding from families you support during the ‘Breastfeeding – a good start together’ project? |  |  |  |  | X |
| Have you had enough time to familiarize yourself with the ‘Breastfeeding – a good start together’-support? |  |  |  |  | X |
| Have you had managerial support in using the ‘Breastfeeding – a good start together’-support? |  |  |  |  | X |
| How do you consider the usefulness of the training programme’s specific topics related to the support of families in line with the ‘Breastfeeding – a good start together’-project?   - Biological background for breastfeeding (including anatomy, physiology, metabolic adaptation, temperature regulation, and suckling technique) - Psychosocial background for breastfeeding (including intention for breastfeeding, self-efficacy, knowledge, support, and social influence) - Breastfeeding as a joint parental task (including father’s/partner’s support, including fathers/partners in the support) - Skin-to-skin contact and the initial breastfeeding - Breastfeeding by demand - Infant thriving signs (including stools, pacifier, supplements, partial breastfeeding, discontinuation of breastfeeding/introducing solids) - Breastfeeding should not hurt – proper positioning of mother and infant during breastfeeding - Working with goals in case of breastfeeding problems (including communication, common breastfeeding problems, and theory-based support) - Providing breastfeeding support uniformly |  |  |  |  | X |
| Have you read the manual included in the ‘Breastfeeding – a good start together’ project? |  |  |  |  | X |
| Yes 🡪 | Did you find reading the manual easy or difficult? |  |  |  | X |
|  | To what extent did you find that the manual contained useful information about working with breastfeeding? |  |  |  | X |
| Overall, to what extent do you feel enabled by the training programme to guide families in line with the ‘Breastfeeding – a good start together’ intervention? |  |  |  |  | X |

^a^ The question about the intervention website (ammeassistenten.dk) was only visible for health visitors in the intervention clusters.
^b^ The question about participation in the training programme were only addressed to health visitors in the control clusters whose managers had informed us had been employed after completion of the baseline questionnaire.
